# Retention of hexanucleotide repeat-containing intron in *C9orf72* mRNA: implications for the pathogenesis of ALS/FTD

**DOI:** 10.1186/s40478-016-0289-4

**Published:** 2016-02-25

**Authors:** Michael Niblock, Bradley N. Smith, Youn-Bok Lee, Valentina Sardone, Simon Topp, Claire Troakes, Safa Al-Sarraj, Claire S. Leblond, Patrick A. Dion, Guy A. Rouleau, Christopher E. Shaw, Jean-Marc Gallo

**Affiliations:** Department of Basic and Clinical Neuroscience, Maurice Wohl Clinical Neuroscience Institute, Institute of Psychiatry, Psychology and Neuroscience, King’s College London, 125 Coldharbour Lane, London, SE5 9NU UK; London Neurodegenerative Disease Brain Bank, Institute of Psychiatry, Psychology and Neuroscience, King’s College London, London, SE5 8AF UK; Montreal Neurological Institute and Hospital, McGill University, Ludmer Building, 1033 Pine Avenue West, Montreal, QC H3A 1A1 Canada

**Keywords:** *C9orf72*, Amyotrophic lateral sclerosis, Frontotemporal dementia, RNA, Repeats, Splicing

## Abstract

**Introduction:**

The most common forms of amyotrophic lateral sclerosis and frontotemporal dementia are caused by a large GGGGCC repeat expansion in the first intron of the *C9orf72* gene. The repeat-containing intron should be degraded after being spliced out, however GGGGCC repeat-containing RNA species either accumulate in nuclear foci or are exported to the cytoplasm where they are translated into potentially toxic dipeptide repeat proteins by repeat-associated non-AUG-initiated (RAN) translation.

**Results:**

In order to determine the mechanisms of repeat-containing intron misprocessing, we have analyzed *C9orf72* transcripts in lymphoblasts from *C9orf72* expansion carriers (*n* = 15) and control individuals (*n* = 15). We have identified polyadenylated *C9orf72* RNA species retaining the repeat-containing intron and in which downstream exons are spliced correctly resulting in a *C9orf72* mRNA with an enlarged 5’-UTR containing the GGGGCC repeats. Intron-retaining transcripts are produced from both wild-type and mutant alleles. Intron-retaining *C9orf72* transcripts were also detected in brain with a 2.7 fold increase measured in the frontal cortex from heterozygous expansion carriers (*n* = 11) compared to controls (*n* = 10). The level of intron-retaining transcripts was increased 5.9 fold in a case homozygous for the expansion. We also show that a large proportion of intron 1-retaining *C9orf72* transcripts accumulate in the nucleus.

**Conclusions:**

Retention of the repeat-containing intron in mature *C9orf72* mRNA can potentially explain nuclear foci formation as well as nuclear export of GGGGCC repeat RNA and suggests that the misprocessing of *C9orf72* transcripts initiates the pathogenic process caused by *C9orf72* hexanucleotide repeat expansions as well as provides the basis for novel therapeutic strategies.

**Electronic supplementary material:**

The online version of this article (doi:10.1186/s40478-016-0289-4) contains supplementary material, which is available to authorized users.

## Introduction

Amyotrophic lateral sclerosis (ALS), a devastating degenerative disease of motor neurons and frontotemporal dementia (FTD), the second most common form of presenile dementia after Alzheimer’s disease, show considerable overlap clinically and genetically. Pathologically, ALS and FTD patients display abundant cytoplasmic inclusions of the DNA and RNA-binding protein, TDP-43, suggesting that both neurodegenerative conditions are likely to represent two ends of a single pathological continuum. The most common forms of familial and sporadic ALS and FTD, referred to as c9ALS/FTD, are caused by a non-coding GGGGCC (G_4_C_2_) hexanucleotide repeat expansion in the *C9orf72* gene on chromosome 9p21 [1, 2]. The number of repeats ranges from 2 to 23 in the normal population but is increased to more than 700–1600 repeats in affected individuals. The proportion of sporadic cases with a G_4_C_2_ repeat expansion depends on geographical origin. For example, in the United Kingdom, the G_4_C_2_ repeat expansion accounts for 20–50 % of familial or 5–10 % of sporadic cases of ALS [3, 4]. The G_4_C_2_ repeat expansion is located either in intron 1, between two 5’-untranslated region (5’-UTR) exons, or in the promoter region according to whether an upstream or a downstream transcription start site is used.

In addition to possible haploinsufficiency, two, not mutually exclusive, mechanisms have been proposed to explain the pathogenesis of c9ALS/FTD: RNA *trans*-dominant toxic effects and repeat-associated non-AUG-initiated (RAN) translation of G_4_C_2_ RNA repeats into potentially toxic dipeptide repeat proteins (DPRs) [5–7]. The G_4_C_2_ repeats are transcribed from both sense and antisense strands of the *C9orf72* gene [5, 8–11]. Sense and antisense *C9orf72* repeat RNA can form nuclear foci that have been detected in brain and spinal cord tissue from affected individuals [1, 5, 9–13] and in neurons differentiated from induced pluripotent stem cells (iPSCs) established from c9ALS/FTD patients [12, 14, 15]. Sequestration of specific RNA-binding proteins by hexanucleotide RNA repeats could impair their RNA processing activity and contribute to pathogenesis. Proteins binding to G_4_C_2_ repeats and co-localizing with foci identified to date include hnRNP A3 [16], Pur α [17], ADARB2 [12], hnRNP H [13, 18] and nucleolin [19]. Sense and antisense RNA repeats can also be exported to the cytoplasm where they are each translated into the three possible reading frames by RAN translation resulting in five DPRs that have been detected in the brain of c9ALS/FTD patients [5–9]. Arginine-rich DPRs (GR, PR) causes neurodegeneration in *Drosophila* [20] or are toxic in transfected cells [21–24].

Impairment of nucleocytoplasmic transport appears to be a key mediator of *C9orf72*-linked pathogenesis. For instance, components of the nuclear pore complex have been identified as modifiers of pathogenesis in several model systems [25–28] and the C9orf72 protein itself binds to components of the nuclear pore complex and its short isoform has been shown to relocalize from the nuclear membrane to the plasma membrane in neurons from expansion carriers [29].

*C9orf72* intron 1, where G_4_C_2_ repeats are located, should be degraded after being spliced out during the processing of pre-mRNA into mature mRNA. However, in c9ALS/FTD, expanded G_4_C_2_ repeats fail to be degraded and form nuclear foci or are RAN translated into DPRs. The mechanisms of defective nuclear degradation as well as of nuclear export of *C9orf72* G_4_C_2_ repeat sequences are presently unknown but are central to the pathogenesis of c9ALS/FTD. Interestingly, treatment with antisense oligonucleotides (ASOs) targeting *C9orf72* exons downstream of intron 1, up to exon 11, promotes degradation of expanded *C9orf72* transcripts [11, 12, 15]. As splicing occurs mainly co-transcriptionally this suggests that intron 1 is still present in nascent transcripts when downstream exons have been spliced. This prompted us to analyze the fate of *C9orf72* intron 1 in cells derived from expansion carriers.

Here we identify polyadenylated *C9orf72* RNA species retaining the repeat-containing intron and in which downstream exons are spliced correctly resulting in a *C9orf72* mRNA with an enlarged 5’-UTR containing the G_4_C_2_ repeats. Generation of intron 1-retaining RNA species potentially explains a number of pathological features of c9ALS/FTD and opens the way to novel therapeutic strategies.

## Materials and methods

### Lymphoblasts

Lymphoblasts from *C9orf72* expansion carriers (*n* = 15) were generated using a standard protocol where the Epstein-Barr virus is used to immortalize B-lymphocytes or were obtained from the UK MND DNA Bank. Control lymphoblasts from individuals free from neurological disease (*n* = 15) were generated as above or were from the European Collection of Animal Cell Cultures (ECACC). Cells have been genotyped for the *C9orf72* G_4_C_2_ hexanucleotide repeat expansion by repeat-primed PCR [1, 2]. Lymphoblasts were grown in RPMI supplemented with 15 % (v/v) FBS, 100 UI/ml penicillin and 100 μg/ml streptomycin.

### Brain tissue

Frozen brain tissue from cases with frontotemporal lobar degeneration (FTLD) or frontotemporal lobar degeneration with motor neuron disease (FTLD-MND) (*n* = 11) and control cases from individuals free from neurological disease (*n* = 10) were obtained from the MRC London Neurodegenerative Diseases Brain Bank (Institute of Psychiatry, Psychology and Neuroscience, King’s College London, UK), and were collected in accordance with local and national research ethics guidelines (Additional file 1: Table S1). The *C9orf72* status of the cases used was confirmed by repeat-primed PCR.

### RNA isolation

Total RNA was extracted from whole lymphoblasts or from cytoplasmic and nuclear fractions (see below) using TRIzol (Life Technologies). Frozen brain tissue samples were homogenized in matrix lysing-D tubes (MP Biomedicals) in conjunction with the Fastprep sample preparation system and total RNA was extracted using the RNeasy lipid tissue kit (Qiagen). RNA from an FTLD case homozygous for the *C9orf72* G_4_C_2_ repeat expansion [30] was obtained from Dr Adrian Isaacs and Dr Pietro Fratta (UCL Institute of Neurology, London). Any residual contaminating genomic DNA was eliminated by treatment with Turbo DNase (Ambion). Poly(A)^+^ RNA was selected from total RNA using oligo(dT) conjugated to magnetic beads (Dynabeads® Oligo(dT)_25_, Ambion) according to the manufacturer’s protocol. RNA concentration was determined with a Nanodrop spectrophotometer (Thermo Scientific). RNA integrity number (RIN) was measured with an Agilent RNA 6000 analyzer (lymphoblasts, controls: 6.0–7.6, expansion carriers: 5.9–7.5; brain, controls: 3.9–6.5, expansion carriers: 3.8–5.7).

### Nuclear and cytoplasmic fractionation

Nuclear and cytoplasmic fractionation was carried out using a modification of earlier protocols [31–33]. Lymphoblasts were centrifuged at 800 rpm for 5 min and lysed by slow pipetting in lysis buffer (10 mM Tris–HCl pH 8.4, 140 mM NaCl, 1.5 mM MgCl_2_, 0.5 % Nonidet P-40, 1 mM dithiothreitol and 100 U/ml RNasin). The suspension was centrifuged at 1000 g for 3 min at 4 °C and the supernatant recovered as the cytoplasmic fraction. Nuclear pellets were resuspended in lysis buffer with 3.3 % (w/v) sodium deoxycholate and 6.6 % (v/v) Tween 40. The samples were vortexed and incubated on ice for 5 min. Nuclei were re-pelleted by centrifugation at 1000 g for 3 min at 4 °C. Pooled supernatants were centrifuged at 1000 g for 5 min at 4 °C and transferred to fresh tubes. Nuclei were lysed in thiocyanate buffer (4 M guanidinium thiocyanate, 20 mM sodium acetate, 0.1 mM dithiothreitol, 0.5 % (w/v) sarkosyl). RNA was extracted from cytoplasmic and nuclear fractions using TRIzol, as above. Final RNA volumes from each fraction were adjusted to represent cell-equivalent concentrations [34].

### RT-PCR

RNA was reverse transcribed using the TaqMan RT kit or the Superscript III kit (Life Technologies) with oligo(dT) or random hexamers according the manufacturer’s protocols. Reverse-transcribed RNA was amplified by PCR using GoTaq polymerase (Promega) using primers and conditions detailed in Additional file 1: Table S2. For PCR across the G_4_C_2_ repeats, reactions were supplemented with betaine (Sigma), DMSO and 7-deaza GTP (New England Biosystems) [1]. No RT controls were used to confirm absence of DNA contamination. RT-PCR products were separated in 1.5 % (w/v) agarose gels and stained with ethidium bromide. The amount of PCR product was estimated by densitometric analysis of the gels using the VisionWorks®LS analysis software (UVP).

### qRT-PCR

For real-time quantitative PCR, reactions contained 5 μl SYBR Green (Roche Diagnostics), 1.25 μM of each primer and 10 ng of cDNA. Primers (Additional file 1: Table S2) were designed using Primer-BLAST. Each primer pair produced a single PCR product and its identity was confirmed by sequencing. To produce standard curves for absolute quantification, PCR products were ligated into the pGEM-T easy vector and transformed into JM109 *E. coli*. Concentrations of plasmid DNA were determined using a Nanodrop spectrophotometer (Thermo Scientific) and copy numbers calculated. Serial dilutions were made to produce standard curves ranging from 10^1^ to 10^7^ molecules (Additional file 1: Figure S5). qPCR was performed in 384-well plates on an Applied Biosystems 7900HT Fast Real-Time PCR System using the conditions detailed in Additional file 1: Table S2. Samples were run in duplicate and the average cycle threshold (Ct) was calculated for target and standards. These values were used to calculate the number of RNA molecules in each sample. Statistical analysis was carried out using the Mann–Whitney *U* test using the SPSS software.

### Sequencing

PCR products were excised from the gels and extracted using the Qiaquick gel extraction kit (Qiagen) and cloned into the pGEM-T Easy vector (Promega) using the TA cloning system. Sequencing of both strands was performed commercially using SP6 and T7 primers (MWG Eurofins). For allele analysis, a 451 bp fragment spanning the intron 1-exon 2 boundary and the rs10757668 SNP was amplified by PCR using standard procedures. Amplicons were directly sequenced with the same primers using Big-Dye Terminator v1.1 and products run on an ABI3130 Genetic Analyzer (Applied Biosystems).

## Results

### Intron 1 retention in polyadenylated *C9orf72* transcripts in lymphoblasts from G_4_C_2_ expansion carriers

Defective splicing of *C9orf72* intron 1 could result in its retention in an otherwise mature mRNA. To determine whether polyadenylated RNA contained sequences derived from *C9orf72* intron 1, we analyzed poly(A)^+^ RNA from cultured lymphoblasts established from heterozygous *C9orf72* G_4_C_2_ repeat expansion carriers and control individuals. Lymphoblasts derived from *C9orf72* G_4_C_2_ repeat expansion carriers display nuclear foci [11], thus, abnormal processing of intron 1 occurring in neurons is likely to be recapitulated in lymphoblasts.

**Fig. 1:**
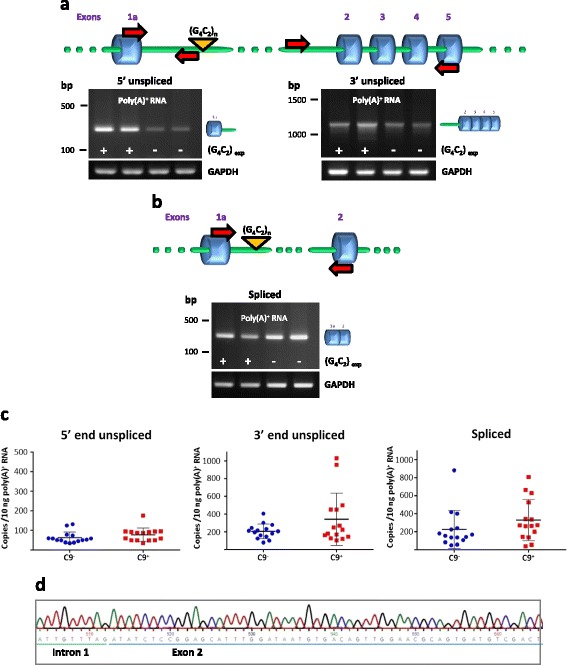
Intron 1 retention in *C9orf72* transcripts in lymphoblasts. *C9orf72* transcripts were analyzed in lymphoblasts from *C9orf72* G_4_C_2_ expansion carriers and control individuals. **a** RT-PCR analysis of poly(A)^+^ RNA using primers spanning the 5’ splice site (left) or the 3’ splice site (right) of intron 1 demonstrating retention of intron 1in polyadenylated RNA. The position of the primers is indicated on the diagram above the gels. GAPDH was used as a loading control. **b** Correctly spliced transcripts detected in controls and expansion carrier cells using primers in exons 1a and 2. **c** Quantitative analysis of intron retention by real-time PCR. Levels of *C9orf72* transcripts spliced or unspliced at the 5’ and 3’ end of intron 1 were determined by real-time qRT-PCR. Data are shown as means ± SEM. Each data point represents an individual case, *n*=15 (C9^-^); 15 (C9^+^). No significant differences were observed between the C9^−^ and C9^+^ groups. **d** Sequencing of the 3’ PCR product demonstrates an exact intron 1-exon 2 boundary. C9^−^, controls; C9^+^, expansion carriers

Poly(A)^+^ RNA was purified from lymphoblasts and reverse transcribed using an oligo(dT) primer. The lack of contaminating genomic DNA was demonstrated by PCR using primers spanning a short intron in the *HOXB4* gene [35] (Additional file 1: Figure S1a). The large size and high GC content of intron 1 prevents its direct amplification by PCR. To determine whether intron 1 sequences were present in polyadenylated *C9orf72* RNA, cDNA was analyzed by PCR using primers spanning the exon 1a-intron 1 and intron 1-exon 2 boundaries. PCR with a forward primer specific for exon 1a and a reverse primer annealing to intron 1, 5’of the G_4_C_2_ repeats, generated a 278 bp product from both control and expansion carrier cells (Fig. 1a, left). PCR with a forward primer annealing to intron 1, 3’of the G_4_C_2_ repeats, and a reverse primer specific for exon 5 generated a 1156 bp product from both control and expansion carrier cells (Fig. 1a, right). Such products were not observed in control reactions with no reverse transcriptase, ruling out the possibility that they originated from residual contaminating genomic DNA (Additional file 1: Figure S2). Oligo(dT) was used as a primer for reverse transcription in order to add a further level of specificity; the same results were obtained when reverse transcription was primed using random hexamers (Additional file 1: Figure S3). Thus, mature polyadenylated RNA species derived from *C9orf72* and containing intron 1 sequences are produced in lymphoblasts from expansion carriers and control individuals. In addition to transcripts retaining intron 1 *C9orf72* transcripts in which intron 1 has been correctly spliced out were also detected in both control cells and cells from expansion carriers (Fig. 1b).

Intron 1 retention was quantified by measuring the levels of *C9orf72* transcripts unspliced at the 5’ end or at the 3’ end by real-time quantitative RT-PCR (qRT-PCR) in lymphoblasts from expansion carriers (*n* = 15) and controls (*n* = 15). No significant differences were detected between the two groups (Fig. 1c). Comparison with normally spliced transcripts shows that about 25 % of *C9orf72* transcripts retain intron 1. This value is comparable with what has been measured for several transcripts in granulocytes [36].

Sequencing confirmed that the 5’ PCR product contained an exact exon 1a-intron 1 boundary and that the 3’ product contained an exact intron 1-exon 2 boundary as well as exon 2–3, 3–4 and 4–5 junctions (Fig. 1d and Additional file 1: Figure S4). The latter product corresponds to a transcript retaining intron 1 and in which the other introns have been spliced out correctly, at least up to intron 4. *HOXB4* analysis and RT negative controls effectively rule out the possibility that the products detected had originated from genomic DNA (Additional file 1: Figures S1a and S2). Furthermore, the 3' end product, that contains exact exon-exon junctions can only have originated from a reverse transcribed RNA.

### Intron 1-retaining transcripts contain the repeat sequence and are produced from both wild-type and mutant alleles

**Fig. 2:**
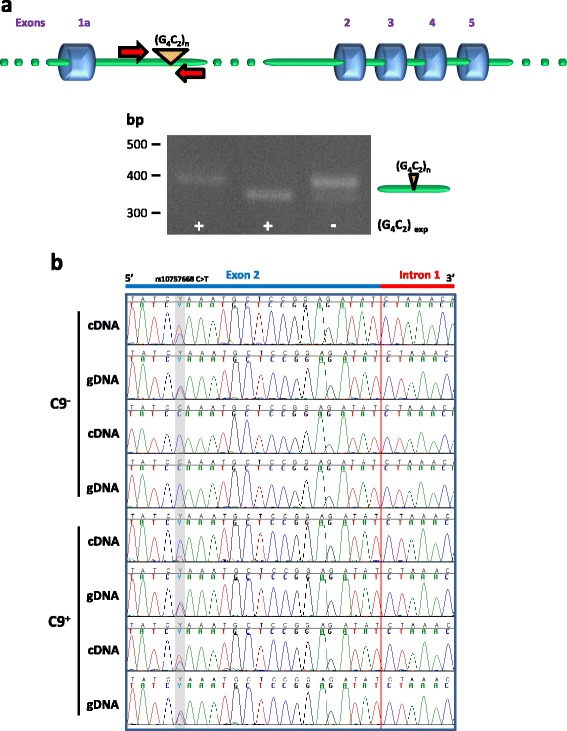
**a** Intron 1-retaining transcripts contain the G_4_C_2_ repeat sequence. PCR using primers spanning the G_4_C_2_ repeat region was performed on cDNA reversed transcribed from polyadenylated *C9orf72* RNA from control and expansion carrier lymphoblasts. The two products of slightly different sizes detected in cells from normal individuals correspond to the two alleles; the single product generated from expansion carrier cells corresponds to the wild-type allele. **b** Intron 1-retaining transcripts are produced from both wild-type and mutant alleles. Sequence traces of *C9orf72* overlapping the intron 1-exon 2 boundary and the rs10757668 SNP in cDNA (top panels) and genomic DNA (gDNA, bottom panels) prepared from lymphoblasts from two control individuals and two expansion carriers heterozygous for rs10757668. One of the control cases (*top trace*) is heterozygous for rs10757668. The sequence of the reverse strand is shown; the position of rs10757668 is indicated by the grey box. Intron 1-retaining transcripts contain both the C and T alleles showing that intron 1 retention occurs in RNA transcribed from the wild-type as well as the expanded allele. C9^−^, controls; C9^+^, expansion carriers

Detection of exact exon 1a-intron 1 and intron 1-exon 2 boundaries in cDNA suggests that the template RNA overlaps the G_4_C_2_ repeat sequence. To confirm that polyadenylated *C9orf72* RNA contained the repeat sequence, we performed a PCR on cDNA using a pair of primers spanning the repeat region. PCR across repeats will only amplify the product from the wild-type allele in expansion carrier cells, as the repeat region from the expanded allele cannot be amplified due to its size and high GC content. Using this method, two products of slightly different sizes were detected in cells from normal individuals and only one product was generated from expansion carrier cells (Fig. 2a). Such products were not observed in control reactions in which reverse transcriptase was omitted, ruling out the possibility that they originated from residual contaminating intronic genomic DNA (not shown). Therefore the repeat region of the two alleles in control cells and of the wild-type allele in expansion carrier cells is present in polyadenylated *C9orf72* transcripts.

To determine whether intron 1-retaining transcripts could be produced from both the wild-type and the mutant allele, we took advantage of the G > A single-nucleotide polymorphism (SNP) rs10757668 in *C9orf72* exon 2 [1]. We first selected two expansion carrier cases heterozygous for rs10757668 from cases that had been genotyped previously [3]. An intron 1-exon 2 fragment overlapping rs10757668 was amplified and sequenced from genomic DNA, as confirmation of heterozygosity for rs10757668, and from cDNA reversed transcribed from poly(A)^+^ RNA. Sequence analysis revealed that intron 1-retaining transcripts contained both the G and A alleles showing that intron 1 retention occurred in RNA transcribed from the wild-type as well as the expanded allele (Fig. 2b).

### Intron 1-retaining *C9orf72* transcripts accumulate in the nucleus

**Fig. 3:**
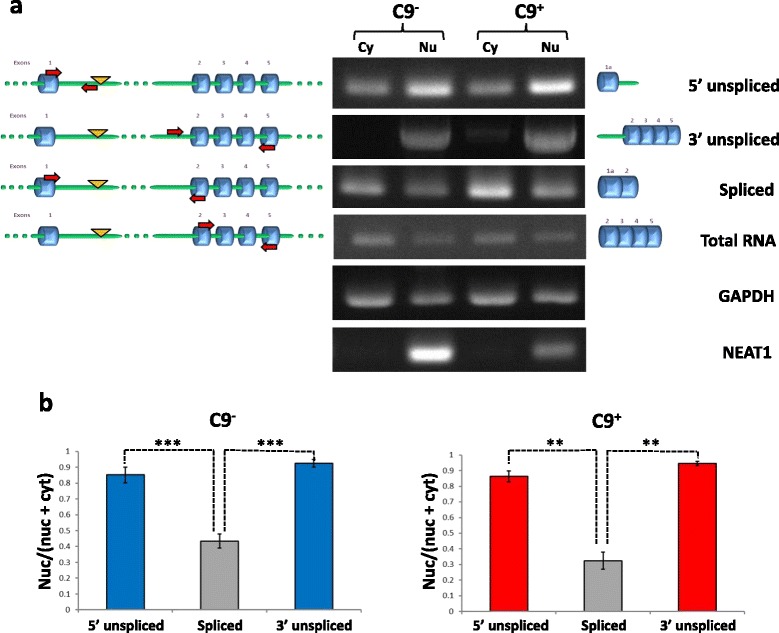
Nuclear accumulation of intron 1-retaining *C9orf72* transcripts. Partitioning of intron 1-retaining and correctly spliced *C9orf72* polyadenylated transcripts was compared between nuclear and cytoplasmic fractions from lymphoblasts from G_4_C_2_ repeat expansion carriers and controls. **a** Poly(A)^+^ RNA was analyzed in nuclear and cytoplasmic fractions by RT-PCR as in Fig. 1. The nuclear long non-coding RNA, NEAT1, was used to confirm the purity of the fractions. GAPDH was used as a loading control. **b** Levels of nuclear and cytoplasmic unspliced and spliced *C9orf72* transcripts were compared according to cell equivalent and the ratios nuclear/(nuclear + cytoplasmic) were calculated for each product. Data represent mean ± SEM, *n* = 6 (C9^+^); 7 (C9^−^), ** *P* < 0.01, *** *P* < 0.001. C9^−^, controls; C9^+^, expansion carriers

A feature of intron-retaining transcripts is their failure to be exported from the nucleus [36, 37]. We therefore compared the partitioning of *C9orf72* transcripts between the nucleus and the cytoplasm in lymphoblasts from G_4_C_2_ repeat expansion carriers and control cases. Nuclear and cytoplasmic fractions were prepared and poly(A)^+^ RNA was extracted from each fraction and reverse transcribed. *HOXB4* analysis confirmed the lack of contaminating genomic DNA in both fractions (Additional file 1: Figure S1b). The purity of the nuclear and cytoplasmic fractions was further assessed by analyzing the polyadenylated nuclear long non-coding RNA, NEAT1 [38]. NEAT1 was only found in the nuclear fraction, demonstrating that the cytoplasmic fraction was free of nuclear contamination (Fig. 3a). Poly(A)^+^*C9orf72* transcripts from both fractions were analyzed for defective splicing of intron 1 at the 5’ and 3’ ends or splicing out of intron 1 (Fig. 3a). Quantification of nuclear and cytoplasmic contents showed that approximately 85 % of *C9orf72* transcripts retaining intron 1 were found in the nuclear fraction whereas correctly spliced transcripts were predominantly cytoplasmic (Fig. 3b). RNA unspliced at the 3’ end and RNA unspliced at the 5’ end were also detected, albeit at low levels, in the cytoplasmic fraction (Fig. 3b).

### Intron 1-retaining *C9orf72* transcripts in brain

**Fig. 4:**
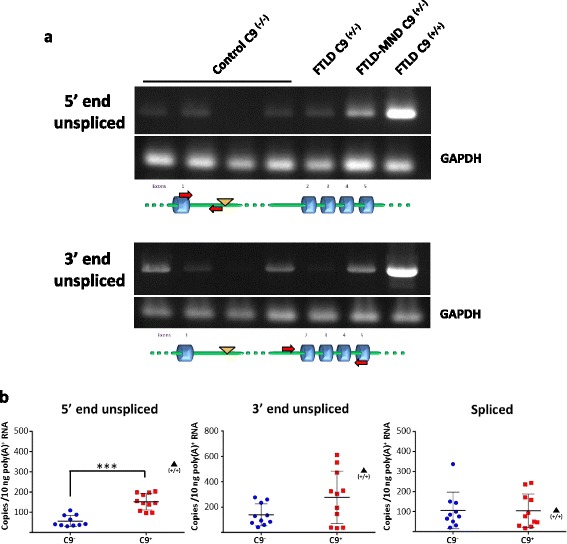
Intron 1 retention in *C9orf72* transcripts in the brain of *C9orf72* expansion carriers. *C9orf72* transcripts were analyzed in the frontal cortex from frontotemporal lobar degeneration (FTLD) or frontotemporal lobar degeneration with motor neuron disease (FTLD-MND) cases with confirmed *C9orf72* hexanucleotide expansions and from control individuals. **a** RNA was analyzed by RT-PCR as in Fig. 1. GAPDH was used as a loading control. The level of *C9orf72* transcripts unspliced at the 5’ and 3’ ends in an FTLD case homozygous for the *C9orf72* G_4_C_2_ repeat expansion was markedly higher than in heterozygous cases (C9^(+/+)^, far right lane). **b** Quantitative analysis of intron retention by real-time PCR. Levels of *C9orf72* transcripts spliced or unspliced at the 5’ and 3’ end of intron 1 were determined by real-time qRT-PCR in heterozygous expansion carriers (*n* = 11) and control cases (*n* = 10). Data are shown as means ± SEM. Each data point represents an individual case, ***P < 0.001, Mann–Whitney *U* test. C9^−^, controls; C9^+^, expansion carriers. ▲(+/+) indicates the values for the single homozygous case analyzed

We next determined whether intron 1-retaining *C9orf72* transcripts were present in brain tissue from *C9orf72* hexanucleotide expansion carriers. RNA from the frontal cortex from heterozygous expansion carriers and from control cases was extracted and analyzed for intron 1 retention. *C9orf72* transcripts unspliced at the 5’ or 3’ end of intron 1 were detected in both control and expansion carriers (Fig. 4a). We also analyzed intron 1 retention in the frontal cortex from an FTLD case homozygous for the *C9orf72* G_4_C_2_ repeat expansion in which results would not confounded by the presence of transcripts from the wild-type allele [30]. The levels of unspliced *C9orf72* transcripts were markedly higher in the homozygous case than in heterozygous cases (Fig. 4a). The levels of *C9orf72* transcripts unspliced at the 5’ end or at the 3’ end were measured by qRT-PCR as for lymphoblasts (Fig. 4b). The level of *C9orf72* transcripts unspliced at the 5’ end in the frontal cortex from heterozygous expansion carriers (*n* = 11) showed a statistically significant 2.7 fold increase compared to control cases (*n* = 10) (Fig. 4b). The level of transcripts unspliced at the 5’ was increased by 5.9 fold in the homozygous case analyzed.

## Discussion

Here, we have identified previously unrecognized RNA species derived from polyadenylated *C9orf72* RNA that are unspliced at the 5’ and 3’ end of intron 1 and in which downstream exons are spliced correctly. As intron 1 is located between two 5’-UTR exons, its retention results in a *C9orf72* mRNA with an enlarged 5’-UTR. Interestingly, higher levels of intron 1-retaining *C9orf72* RNA species were detected in the frontal cortex of expansion carriers compared to control individuals. Two recent papers also reported increased levels of sense and antisense *C9orf72* RNA containing intron 1 in the frontal cortex from expansion carriers [6, 39]. However, these reports did not address the molecular nature of the RNA species containing intron 1 that accumulate in disease. Intron-containing RNA can have various origins; for example, it could represent incompletely processed pre-mRNAs stalled during the splicing process, splice-defective lariat intermediates or aborted transcripts [19, 40]. Here we demonstrate that intron 1-containing *C9orf72* RNA that accumulates in the brain from expansion carriers is, at least in part, a fully processed mRNA retaining intron 1 within an enlarged 5’-UTR. This has important mechanistic implications for the pathogenic process, as discussed below. No significant difference was found in the levels of transcripts unspliced at the 3’ end of intron 1 in brain. This could be explained by the fact that RNA unspliced at the 5’ end of intron 1 originates from transcription variants that contains the hexanucleotide repeat sequence, whereas RNA unspliced at the 3’ end of intron 1 can, in addition, originate from transcription variants not containing the repeat sequence.

Intron 1 retention is consistent with the degradation of expanded repeat-containing transcripts induced by RNase H-sensitive ASOs targeting *C9orf72* RNA up to exon 11 [11, 12, 15]. As splicing out of introns occurs, in most cases, early during transcription, intron 1 is likely not to have been spliced out when transcription has reached exon 11. The presence of the expansion in a mature mRNA in patient tissue is also consistent with a recent report showing that the repeat expansion has to be in the context of an mRNA to cause toxicity in *Drosophila* [41].

Intron 1-retention was observed in *C9orf72* polyadenylated RNA from both the wild-type and the expanded alleles and contains the G_4_C_2_ repeats , and, thus, is part of a normal process. This is not an unusual situation as intron detention (delayed splicing) or retention, either arising from defective splicing or as a regulatory process, is a common occurrence within the mammalian transcriptome [36, 37, 42, 43]. Although retention of intron 1 in *C9orf72* transcripts appears to be independent of the presence of an expanded G_4_C2 repeat sequence, this process results in the expanded G_4_C_2_ sequence from the mutant allele being included in the 5’-UTR of a fully processed mRNA. *C9orf72* mRNA with an enlarged 5’-UTR that includes the G_4_C_2_ repeats, is similar to *FMR1* transcripts associated with fragile X-associated tremor ataxia syndrome (FXTAS), caused by moderate (<200) CGG repeat expansions in the 5’-UTR of the *FMR1* gene [44].

In addition to transcripts retaining the entire intron 1 our analysis does not rule out the presence of shorter RNA species, for example resulting from the use of cryptic polyadenylation sites in intron 1. Use of cryptic intronic polyadenylation sites has been reported in Huntington’s disease, caused by a CAG repeat expansion in exon 1 of the *HTT* gene, resulting in a truncated, aggregation prone, protein [45].

No difference in the level of intron retention was detected between lymphoblasts from expansion carriers and controls. By contrast *C9orf72* transcripts unspliced at the 5’ end of intron 1 were found to be expressed at higher levels in the frontal cortex of expansion carriers compared to control individuals. This was particularly clear in the brain of a homozygous case albeit material from one case only was available for analysis. As suggested by Mori et al*.* [6] the increased levels observed could reflect stabilization of expanded intron 1-containing transcripts. Alternatively, long G_4_C_2_ sequences could also reduce splice site usage, hence inhibiting intron 1 splicing. Of note, introns with a high GC content are prone to be retained [36]. Partial retention of expanded CCTG repeat-containing first intron of the CCHC-type zinc finger (*CNBP*, formerly known as *ZNF9*) gene has also been reported in myotonic dystrophy type 2 (DM2) [46]. Long G_4_C_2_ repeat tracts might also slow down the rate of transcription of the *C9orf72* gene in expansion carriers, hence promoting intron retention.

We found that a large proportion of intron 1-retaining *C9orf72* transcripts accumulated in the nucleus. Nuclear retention of incompletely spliced transcripts has been demonstrated as a regulated process for the control of gene expression or as part of a surveillance pathway (for reviews see [47–49]). Consistent with this notion, intron-containing transcripts resulting from defective splicing in heat-shocked cells are retained in the nucleus [37]. Nuclear accumulation of *C9orf72* mRNA retaining intron 1 could be the consequence of disrupted nucleocytoplasmic transport of mRNAs resulting from *C9orf72* repeat expansion toxicity [26, 50]. However, this is unlikely to be the case as correctly spliced transcripts (e.g. spliced *C9orf72*) are prevalent in the cytoplasm. Of note, huntingtin mRNA with expanded CAG repeats accumulates in the nucleus [51].

**Fig. 5:**
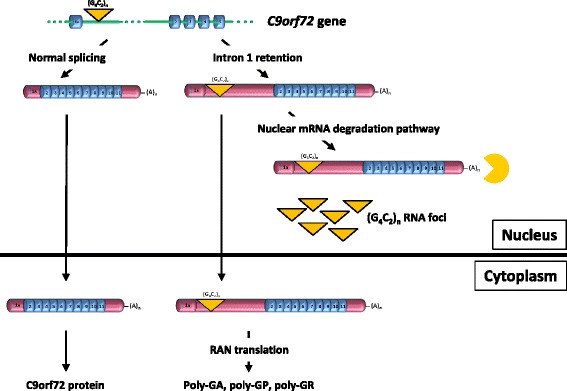
Model of expanded *C9orf72* transcripts processing explaining the main pathological features of c9ALS/FTD. Retention of intron 1 generates a *C9orf72* mRNA with an enlarged 5’-UTR containing the G_4_C_2_ repeat sequence. The majority of intron 1-retaining *C9orf72* mRNA accumulates in the nucleus where it is targeted to a specific degradation pathway unable to process G_4_C_2_ RNA repeats that subsequently aggregate into foci. A small proportion of intron 1-retaining *C9orf72* mRNA is exported to the cytoplasm for RAN translation into DPRs

mRNAs that fail to be exported to the cytoplasm are degraded in the nucleus by a surveillance pathway that has been studied in yeast and reported, but yet to be fully characterized, in mammals [52, 53]. This pathway may involve poly(A)-binding proteins (PABPs) and nuclear exosomes [54–56]. Intron 1-retaining *C9orf72* mRNA may be targeted to such a pathway that would be unable to destabilize the G-quadruplex structure formed by the G_4_C_2_ repeat sequence [19, 57–59] and the repeat region would be protected from degradation, resulting in its accumulation in foci (Fig. 5). While the above pathway may be the pathway to which intron-retaining *C9orf72* mRNA is targeted, it is important to note that RNA can be degraded through different pathways. Indeed, as mentioned above, long G_4_C_2_ repeat sequences can be degraded following ASO-mediated RNase H cleavage of *C9orf72* RNA [11, 12, 15].

Although the majority of intron 1-retaining *C9orf72* transcripts accumulate in the nucleus, intron retention could also explain export of the expanded G_4_C_2_ repeat RNA to the cytoplasm where it would become template for RAN translation into DPRs. As intron 1-retaining *C9orf72* transcripts have the structure of mature mRNAs, an, albeit small, proportion is exported to the cytoplasm through the conventional pathway of nuclear export of mRNA (Fig. 5). However, this does not exclude that other G_4_C_2_ repeat-containing RNA species might be the main template for RAN translation.

Finally, intron retention in the sense transcript might explain transcription of repeats from the reverse strand. G_4_C_2_ expansions in the *C9orf72* gene have been shown to promote the formation of RNA-DNA hybrids (R-loops) [19]. Intron retention as well as formation of R-loops could be linked to the slowing down of transcription of the sense transcript by the repeat sequence [60]. R-loops would, in turn, induce antisense transcription [60].

## Conclusions

We have identified *C9orf72* mRNA species with an enlarged 5’-UTR that includes the G_4_C_2_ repeat sequence that can explain a number of features of c9ALS/FTD. Interfering with intron 1 processing would offer novel ways of dissecting the cascade of events leading to neuronal dysfunction and death and represents an innovative and promising therapeutic strategy for c9ALS/FTD.

## Additional file

Additional file 1: **Supplementary material.** (PDF 456 kb)
