# Supplementary material for: Retention of hexanucleotide repeat-containing intron in C9orf72 mRNA: implications for the pathogenesis of ALS/FTD
Source: Acta Neuropathol Commun. 2016 Feb 25;4:18. doi: 10.1186/s40478-016-0289-4 (PMC4766718; doi:10.1186/s40478-016-0289-4)
Supplement: Supplementary file 1 — Supplementary material. (PDF 456 kb) [file 40478_2016_289_MOESM1_ESM.pdf]

SUPPLEMENTARY MATERIAL

	Expanded cases (n = 12)	Controls (n = 10)
Gender (male)	6 (50%)	3 (30%)
Age at death (years)	59 (39 - 71)	81.3 (38 - 90)
RIN frontal cortex	4.9 (3.8 - 5.7)	4.6 (3.9 - 6.5)
Diagnosis		
FTLD	1 (8%)	-
FTLD/MND	3 (25%)	-
MND	8 (66%)	-

Supplementary Table 1 Demographic, clinical and biochemical data of brain samples used for qRT-PCR analysis. FTLD, frontotemporal lobar degeneration; FTLD-MND, frontotemporal lobar degeneration with motor neuron disease.

SEQUENCE	LOCATION	NAME	INITIAL SEPARATION	CYCLE NO.	DENATURATION	ANNEALING	EXTENTION	FINAL EXTENTION
For GGTGCGTCAAACAGCGACAAGTTC	Exon 1a	*C9orf72* 5' primer pair	5 min, 95°C	36	1 min, 95°C	40 s, 61°C	30 s, 72°C	10 min, 72°C
Rev GCTACAGGCTGCGGTTGTTTCC	Intron 1							
For GGACACAATTCAACCCACAAGTG	Intron 1	*C9orf72* 3' primer pair	5 min, 95°C	37	1 min, 95°C	40 s, 64°C	1 min, 72°C	10 min, 72°C
Rev GCCTTCATGACAGCTGTCACC	Exon 5							
For GCAAGAGCAGGTGTGGGTTTAGG	Exon 1a	*C9orf72* intron 1 splicing out	5 min, 95°C	35	1 min, 95°C	50 s, 60°C	50 s, 72°C	10 min, 72°C
Rev CATCTATAGCACCACTCTCTGC	Exon 2							
For GCAAGAGCAGGTGTGGGTTTAGG	Exon 1a	*C9orf72* repeats spanning	5 min, 95°C	40	1 min, 95°C	1 min, 59°C	40 s, 72°C	10 min, 72°C
Rev AGTCGCTAGAGGCGAAAGC	Intron 1							
For GCACCTACTGTGCTAGTTGAATGTC	Intron 1	*C9orf72* exon 2 SNP detection	5 min, 95°C	35	1 min, 95°C	1 min, 55°C	1 min, 72°C	10 min, 72°C
Rev CATCTATAGCACCACTCTCTGC	Exon 2							
For GCAGAGAGTGGTGCTATAGATG	Exon 2	Total *C9orf72*	5 min, 95°C	25	1 min, 95°C	30 s, 60°C	40 s, 72°C	10 min, 72°C
Rev GCCTTCATGACAGCTGTCACC	Exon 5							
For GTGGACCTGACCTGCCGTCTAG	Exon 7	*GAPDH*	5 min, 95°C	25	1 min, 95°C	30 s, 61°C	20 s, 72°C	10 min, 72°C
Rev CCTGTTGCTGTAGCCAAATTCGTTG	Exon 8							
For CCTGGTCTTGTGGAACTGAACTTAGC	NEAT1 lncRNA	*NEAT1*	5 min, 95°C	28	1 min, 95°C	30 s, 59°C	30 s, 72°C	10 min, 72°C
Rev TAAAGCGTTGGTCAATGTTGTCC								
For CTACCCCTGGATGCGCAAAG	Exon 2	*HOX4B*	5 min, 95°C	35	1 min, 95°C	50 s, 61°C	1 min, 72°C	10 min, 72°C
Rev CGAGCGGATCTTGGTGTTGG	Exon 3							
**Conditions for qRT-PCR**								
For GCAAGAGCAGGTGTGGGTTTAG	Exon 1a	*C9orf72* 5' primer pair	10 min, 95°C	N/A	30 s, 95°C	25 s, 61°C	25 s, 72°C	N/A
Rev CCTCAGCGAGTACTGTGAGAGC	Intron 1							
For CCTGATAGGAGATAACAGGATTCCAC	Intron 1	*C9orf72* 3' primer pair	10 min, 95°C	N/A	30 s, 95°C	25 s, 61°C	25 s, 72°C	N/A
Rev CGACATCACTGCATTCCAACTGTC	Exon 2							
For GCAAGAGCAGGTGTGGGTTTAG	Exon 1a	*C9orf72* intron 1 splicing out	10 min, 95°C	N/A	30 s, 95°C	25 s, 61°C	25 s, 72°C	N/A
Rev CGACATCACTGCATTCCAACTGTC	Exon 2							

Supplementary Table 2 Primer sequences and PCR conditions.

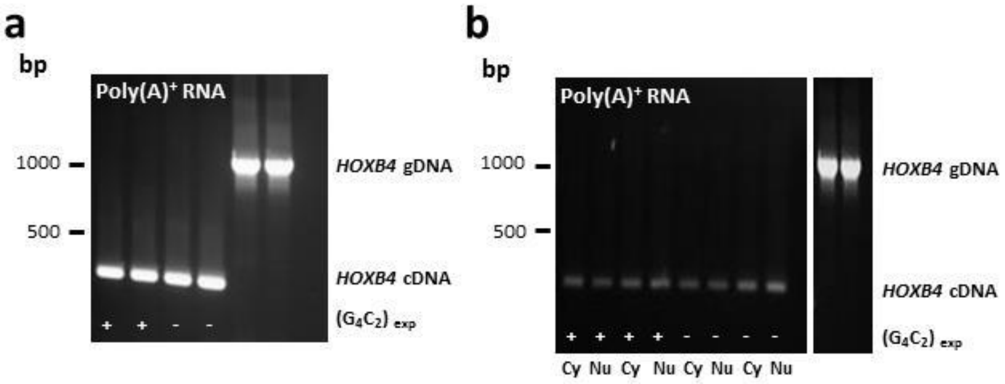

Supplementary Fig. 1 a PCR analysis of *HOXB4* confirming the absence of genomic DNA (gDNA) contamination in cDNA reversed transcribed from poly(A)$^+$ RNA purified from lymphoblasts. PCR was performed using primers flanking an 842 bp intron of the *HOXB4* gene. A single 268 bp product, containing exonic sequences but lacking the intron was detected in cDNA samples from lymphoblasts whereas an 1110 bp product, containing exonic sequences and the intron was detected in control gDNA. **b** Absence of contaminating genomic DNA in cytoplasmic and nuclear fractions. PCR analysis of *HOXB4* confirming the absence of genomic DNA contamination in cDNA reversed transcribed from poly(A)$^+$ RNA purified from nuclear and cytoplasmic fractions from expansion carrier and control lymphoblasts. PCR analysis was performed as described for Fig. 1.

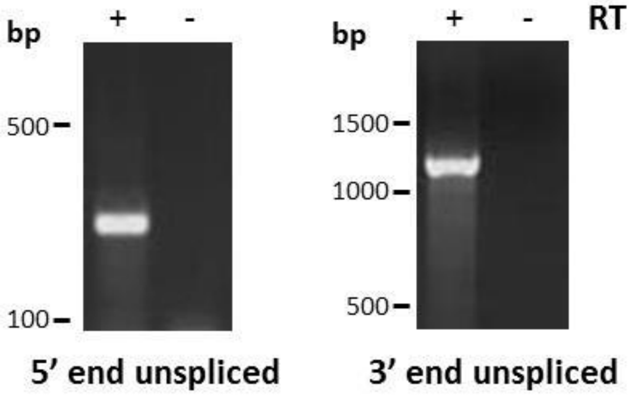

Supplementary Fig. 2 RT-PCR analysis of poly(A)$^+$ RNA isolated from lymphoblasts using the 5' primer pair or the 3' primer pair as in Fig. 1, with or without reverse transcriptase.

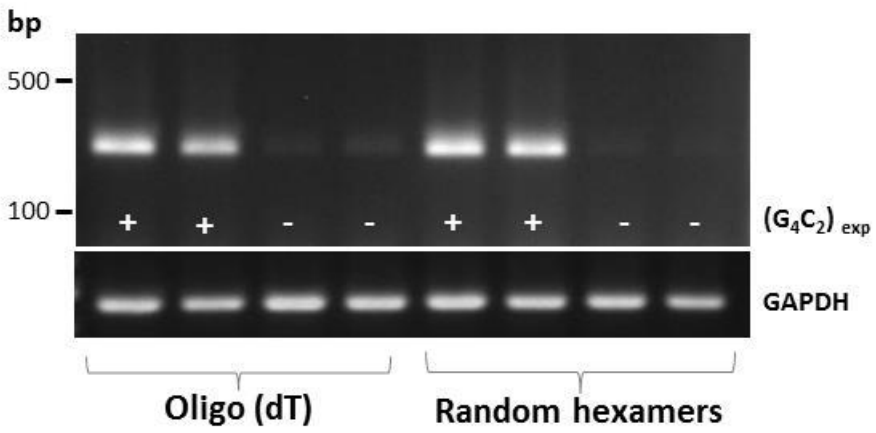

Supplementary Fig. 3 Comparison of reverse transcription priming with oligo(dT) and random hexamers. PCR was performed with the 5’ primer pair, as described for Fig. 1.

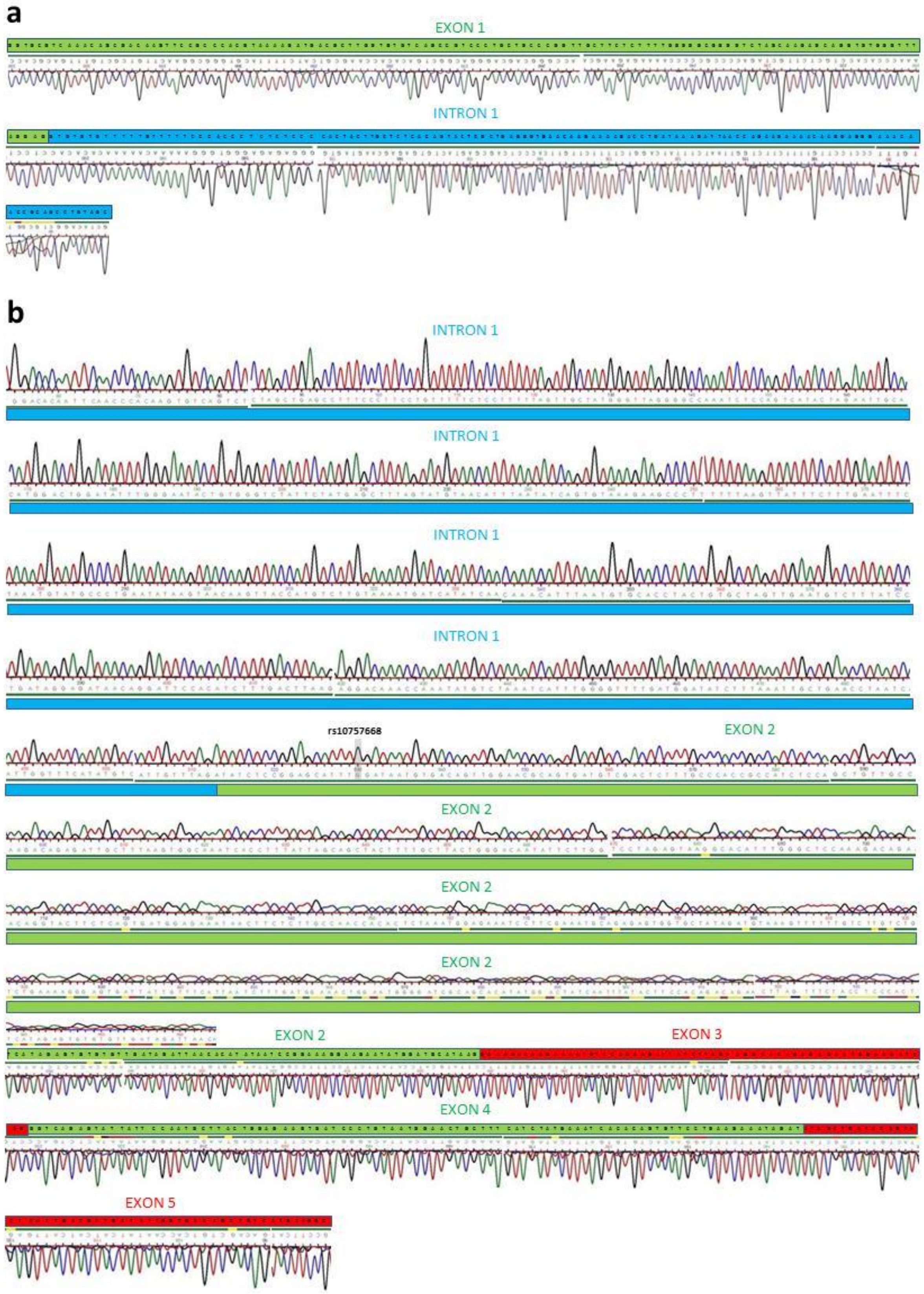

Supplementary Fig. 4 Sequence of intron 1-retaining *C9orf72* transcripts. **a** Chromatogram of the sequence of the PCR product obtained with the 5’ primer pair showing the exon 1a-intron 1 boundary. The sequence of the reverse strand is shown. **b** Chromatogram of the sequence of the PCR product obtained with the 3’ primer pair showing the intron 1-exon 2 boundary and exon 2-3, 3-4 and 4-5 junctions. The sequence of the reverse strand is shown for the 3’ end of the product.

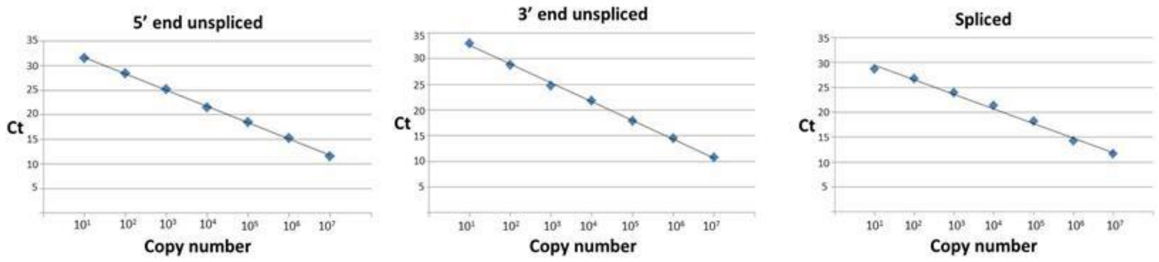

Supplementary Fig. 5 qPCR standard curves for the determination of levels of *C9orf72* transcripts unspliced at the 5' end or 3' end of intron 1, or spliced transcripts.
